# Observations on the Pathology of Croup: With Remarks on Its Treatment by Topical Medications

**Published:** 1849-02

**Authors:** 


### REVIEWS.

ART. IX.—*Observations on the Pathology of Croup:* with remarks on its Treatment by Topical Medications. By HORACE GREEN, A.M., M.D., etc., etc. New York: John Wiley, 161 Broadway, and 13 Paternoster Row, London. 1849. 16mo. pp. 115.

The first chapter of this little work is devoted to a brief discussion of the Nature and Pathology of Croup. After a thorough examination of the various doctrines which have been propounded by the multitude of writers on this subject since the year 1807, when Napoleon issued his order "d'ouvrir un concours sur la maladie connue sous le nom de croup," and after much experience in and observation of the disease for the last twenty years, the author has come to the following conclusions:

"1. That true croup, pathologically considered, is a special or single disease; being dependent for its existence, like tubercular phthisis, on a peculiar or specific cause.

"2. That its distinctive and essential characteristics consist in an inflammation of the secreting surfaces of the fauces, larynx, and trachea, which is always productive of a membranaceous or an albuminous exudation.

"3. That the membranaceous concretion, which is found coating the inflamed mucous surface of the parts in croup, is an exudation,—not from the membrane itself, but is secreted by the muciperous glands, which so abundantly stud the larynx and trachea.

"4. That the exudative inflammation commences invariably in the superior portion of the respiratory passages, and extends from above downwards—never in the opposite direction."

The truth of the first proposition, it seems to us, cannot be questioned. It follows as a natural and inevitable consequence of the second; for if the distinctive and essential characteristics of the disease consist in the inflammation of a given part, which inflammation is always attended by a certain product, it would surely be absurd to say that another inflammation unattended by this product, is the same thing. The very definition of croup therefore necessarily implies the idea of unity. There may, it is true, be *varieties* due to "difference in constitution, epidemic influences and other circumstances;" and our author very properly, we think, makes three divisions; viz: "*laryngeal*, *tracheal*, and *bronchial*, according to the seat of the greatest intensity of inflammatory action."

*Spasmodic Croup* is used by different persons to express two very different conditions of the upper portion of the air passages. By some, the term is applied to simple spasm of the glottis unaccompanied by inflammation and effusion of fibrin. This is a most incorrect application of the word croup.

But there is, properly speaking, a spasmodic variety of this disease. When it affects "children of nervous temperament or weak and irritable habits," it often assumes a spasmodic form. In all these cases, nevertheless, according to the observations of Guersent and others, lymph is thrown out, and "albuminous concretions—sometimes extensive, but more frequently consisting of small isolated patches—are found in the larynx." These exudations are not at first sufficient in quantity to offer much obstruction to respiration; when, therefore, the spasm subsides, there will be an intermission, in which the breathing is comparatively free and easy. As however, the inflammation progresses, and the exudation increases in quantity, the spasms will become more and more frequent in their occurrence, and the difficulty of breathing in the intervals, greater and greater.

The second proposition (leaving out the word 'fauces,' of which more presently) is but the almost, if not altogether, unanimous opinion of the best pathologists of the present day. This exudation will not always, by any means, be firm and concrete, moulded as it were to the cast of the tube it occupies, a true false membrane; for sometimes there may be only flakes or shreds of fibrin intermixed in small quantities with mucus; or again, it will be in the form of a layer "resembling, both in color and consistency, that which settles upon scalded cream." There may be these or other differences, but in some form or other fibrin will always be found in the larynx or trachea, or both—the quantity and plasticity depending on the intensity of the inflammation, the robustness of the patient's constitution, etc., etc.

With respect to the third point, viz: the *source* of this exudation, there is no such unanimity. Dr. Green supports his opinion by reference to anatomy, asserting that the lining membrane of the larynx is studded with mucous follicles; that the superior vocal cords and the ventricles of the larynx are full of them, and the trachea still more abundantly supplied. He insists that "it will be found that wherever these glands are the most numerous, there, *cœteris paribus*, will the albuminous exudation be most abundantly poured out, and the adventitious membrane will be the densest and most perfectly formed:" adducing also the testimony of Prof. Hasse, who says that "filamentous bands are sometimes found between the plastic exudation and the mucous membrane, consisting merely of delicate fibrous threads which dip into the orifices of the muciparous glands."

These facts, if all be such, render the correctness of his views highly probable if they do not place them beyond a doubt. This opinion, however, is by no means original with Dr. Green.

We must confess that we cannot help suspecting that the author's uncompromising adherence to the fourth proposition is due not a little to theory; in other words that he arrived at this conclusion rather from the results of his mode of treatment than from actual observation. For soon after stating it, he goes on to say—

"Let this important point, advanced in this last proposition, with reference to the pathology of the disease, be fully established, and universally understood by the profession, and it will be at once perceived what important results may follow the topical employment of appropriate remedial agents, in the early treatment of true inflammatory croup."

Be this as it may, we cannot assent wholly to its truth. If he adduce the names of Professors Rokitansky and Hasse in support of his views, we meet him with those of Porter, Stokes, Condie, and Guersant, and a host of others too numerous to mention, who, while they admit that in the majority of cases the inflammation progresses from above downward, positively assert that such is not always its course.

Other points of interest, such for instance as the influence of age as a predisposing cause, the post-mortem appearances, the nature of the false membranes, etc., are briefly but clearly noticed under this head. The author then proceeds to give some cases illustrative of his mode of treatment and its results.

We are extremely glad to see that the author has, with great candor, related some of the unsuccessful, as well as the more successful cases. It is too much the habit of medical men in reporting the results of their practice to mention only those cases which had a fortunate issue, fearful, we suppose, that their reputation might be injured, or their skill impugned, or their treatment condemned, if they suffered the world to know that a patient had died in their hands, and not seeming to be aware that any save themselves have the slightest interest at stake. Such conduct cannot be too severely reprehended.

We copy one or two of Dr. Green's cases. They will serve as a fair sample of the whole.

"On the evening of the 20th of November, 1842, I was called to see John S——, aged three years, the son of a widow woman in this city. He was in the last stage of croup, having been attacked with the disease about a week previous to the time of my being called. Catarrhal symptoms had preceded, for several days, the full development of the croupal stage.

"The ordinary remedies had been employed, but without arresting in any degree the progress of the disease. The great prostration, the stridulous respiration, and other symptoms of threatened suffocation, which were present, indicated the stage of collapse, and that no relief could be expected from the employment of common means. Under these circumstances, I proposed the cauterization of the larynx, with the hope that some relief might be obtained from this operation.

"The proposition being acceded to by the friends of the little sufferer, I proceeded at once to apply a solution of the nitrate of silver, of the strength of twenty grains to the ounce of water, freely to the fauces, and into the cavity of the glottis. The application was followed by a violent expulsive cough; by which a large quantity of ropy mucus was discharged. Considerable relief followed this operation; the respiration became much less embarrassed, and so continued during a greater part of the following night. Towards morning, however, the croupal symptoms recurred with much violence, and when I saw my patient at an early hour the next day, the prognosis appeared so unfavorable that I did not deem it advisable to renew the application.

"The patient died the same day. No examination of the body was made.

"This case is adduced as being the first instance, in my practice, where a successful attempt had been made to introduce the nitrate of silver into the larynx of a child affected with croup. During a period of nearly four years, previous to this time, as may be seen by referring to my work on diseases of the air passages, I had been constantly using this remedy, locally, in chronic laryngeal and bronchial diseases of adults.

"But up to this period, such were the prejudices against its employment, and such the scepticism of a large proportion of the profession on the subject of topical applications to the larynx in cases of adults even, that hitherto I had not ventured upon its use in the treatment of disease in young children. The marked relief which for a time followed its employment in the above case, although adopted as the "ultimum remedium," in the hopeless stage of the disease, and the small amount of irritation caused by the application, encouraged me to repeat the remedy on subsequent occasions.

"At a late hour in the evening of the 23d February, the Rev. Dr. B., of this city, called at my office and desired me to accompany him, to see his little daughter, a child three years of age, who that evening had been violently seized with an attack of croup.

She had been hoarse, and had had a rough, dry cough for several days previous to the full development of the affection. When I saw her a few hours after the occurrence of the disease, the symptoms of croup, which were present, were marked and severe,—indeed, as I entered the hall of the house, the ringing cough and stridulous respiration of the child—sounds which no physician ever desires to hear a second time, were distinctly audible through the closed doors of the chamber above.

"With the aid of Dr. J. Hancock Douglas, who was in my office when the father of the sick child called, and who accompanied me to his house, I succeeded in obtaining a good view of the throat of the little patient. The parts were highly inflamed, and the tonsils were covered with an albuminous exudation. The barking cough and the embarrassed and tracheal respiration plainly indicated the stage of the disease, and that the inflammation had extended to the larynx, and about the vocal chords.

"As no time was to be lost, I immediately administered ten grains of ipecacuanha, and after waiting fifteen minutes, prepared to cauterize the diseased parts.

"Assisted by Dr. D., I applied a solution of the nitrate of silver (forty grains to the ounce) to the fauces and pharynx, and also introduced the sponge saturated with the solution, into the cavity of the larynx. The introduction of the instrument was followed by a free discharge of muco-fibrinous matter, in which, and also on the sponge, were shreds of the membranous deposit.

"The little patient, very soon after the operation, appeared greatly relieved.

"We remained nearly an hour after the application, in order to repeat it if the symptoms should indicate its necessity; but the child continuing to improve, we left for the night, after giving directions to have an emetic of ipecacuanha administered should there be any increase of the embarrassed respiration. Soon after we left the child fell asleep, and although the breathing was labored, and the cough, which occurred often during the night, was croupal, yet she slept for several hours, and when I called the next morning, I found a great improvement in all the above symptoms. The emetic had not been given. As there still appeared to be some inflammation about the throat, and the cough retained the peculiar sound of the disease, I had fears that there might be a return of all the unfavorable symptoms before night, and therefore concluded not to wait, but to repeat the application of the nitrate of silver to the diseased organ. This was done, and I feel confident the operation was attended with much advantage, for instead of having a recurrence of the croupal symptoms on the second night, as had occurred in a former instance, and which is so likely to follow a remission of the disease in most cases, there was a constant improvement of all the croupal symptoms during that day, and the following night was passed with equally favorable indications.

"In short, after this date no further medication was needed, for the child rapidly recovered."

This last case comes appropriately under the head of *laryngeal* variety. Of the *bronchial* variety take the following:

"In the early part of the spring of the present year, M. M. of this city called on me, and requested me to visit his little daughter, who, under the care of his family physician, had been sick with the croup for nearly a week, and was then dangerously ill.

"One or two of his children had already died of croup, and the array of symptoms which I found here presented, indicated an equally unfavorable termination of the disease in the case of this child. From the history given, as well as from the symptoms present, I found that bronchial inflammation had become complicated with the croupal affection soon after the attack. The symptoms present at this stage of the disease were not dissimilar to those that existed in the latter stage of the case last recorded. There was oppressed and stridulous respiration; the voice was reduced to a whisper; the characteristic croupy cough was present, but more suffocative and bronchial than when occurring in simple croup; and complicated with these symptoms were further indications of extensive bronchial disease. Throughout the left lung especially, of this patient, the sibilant respiration and other evidences of the affection were most apparent.

"The ordinary general treatment usually adopted in such cases had been judiciously and perseveringly employed for several days by the attending physician, apparently without arresting in any degree the progress of the disease.

"I proceeded at once to cauterize the diseased organs, and having applied a strong solution of the nitrate to the fauces, pharynx, and about the glottis, passed the sponge well filled with the fluid into the cavity of the larynx. As had occurred in other cases, this operation was followed by a free expectoration of muco-purulent matter, large quantities of which adhesive discharge were wiped from the mouth of the patient in which, and adhering also to the sponge of the probang were many small fragments of the false membrane.

"These first applications were made about four o'clock in the afternoon; at eleven o'clock at night they were repeated, when other portions of the adventitious membrane were ejected; and within half an hour after the last application there was a decided improvement in all these unfavorable symptoms.

"As the child was greatly enfeebled, not only by the severity of the disease, but from the energetic practice which had been employed, a stimulating expectorant was the only remedy ordered; anodyne and slightly irritating fomentations were applied to the chest; and a bland, supporting diet was directed.

"On calling at an early hour the next morning, the attendants reported the patient as having passed a better night than had occurred to her since the first attack of the disease; and the appearance of the child indicated a favorable change in all the unpromising symptoms. The breathing was much less embarrassed, the pulse and respirations were diminished in frequency, and the cough had nearly lost its croupy character.

"The same plan of treatment was continued, and from this period the patient recovered rapidly; and although it was several days before the voice was restored, yet vocalization returned, and the child was ultimately restored to robust health."

Then follow one or two cases illustrating the spasmodic variety, reported by himself, and some by Dr. Bryan, of Philadelphia, Dr. Blakeman, of New York, and Dr. Ware, of Boston, treated in a similar manner and with like success.

This treatment of croup by the local application of nitrate of silver is not original with Dr. Green. First recommended by Mackenzie, it has since been advocated by MM. Gérouard, Trousseau, Guersant, and other

French physicians. Bretonneau used a stick of whalebone, properly curved, having a small piece of sponge attached to its extremity. This being dipped in a weak solution of nitrate of silver is carried to the back part of the mouth, and a few drops are squeezed into the larynx. Trousseau uses, we believe, a small syringe, which is passed through the glottis and emptied of its contents. Dr. Green prefers a whalebone rod, tipped with sponge, which he introduces into the cavity of the larynx, thus surely and certainly making a direct application to the diseased parts. The strength of the solution he uses is from ℈ij to ℈iv to water ℥j. Of the perfect feasibility of this procedure he is satisfied, because he and others have repeatedly practiced it; and of its value and utility he is no less satisfied, because he has often witnessed the happiest effects, and has yet to see one single instance of any injury in any way resulting from it. If his testimony is to be relied upon, and we see no reason why it may not be, we are bound to admit that he deserves many thanks from his professional brethren, as well as the community at large. Croup is one of the most distressing maladies with which the physician has to deal, and anything which promises to mitigate its terrors deserves an ample and impartial trial before it is condemned. Such it is earnestly to be hoped will be the case in the present instance.

Nitrate of silver, it is well known, exerts a most powerful, and indeed almost magical control over other inflammations of the mucous membranes, so that we have both reason and experience urging us to make a farther trial of this remedy.

Dr. Green was in the habit, as will be seen by a reference to his "Treatise on Diseases of the Air Passages," of treating inflammations of the larynx in adults by topical medication, for several years before he tried them at all in children, and with uncommon success. As before remarked, he lays no claim to originality in the principle of such medications; but he deserves credit if not for improvement in the application of the principle, at least for the perseverance with which he has used them, and the success of the practice in his hands; as well as for the zealous and earnest manner in which he has endeavored to satisfy all as to the truth of his views and the efficacy of this method of treating such diseases.

Whilst laying so much stress on the local use of nitrate of silver, he by no means discards what are recognized as the standard remedies. Emetics receive their due consideration. Whatever revolutions experience may make in the treatment of various diseases, emetics can never be dispensed with in the management of croup. From the time when the disease was first known down to the present day, they have formed the sheet-anchor of hope. Nor can we join with our author in his somewhat sweeping allegations respecting the danger of tartar emetic.

*Bloodletting.*—This remedy though strongly advised by many authors, is as strongly condemned by Bretonneau and other distinguished physicians. Dr. Green is by no means a warm advocate for it, but still "would not altogether discard" it. In strong and plethoric children in the earliest stage of the disease, it will be often of great utility. Under other circumstances it had best be let alone. Calomel in all; and hydrocyanic acid in some cases, is highly recommended.

We here take leave of our author and of his little work, with the expression of the hope that it will be read and pondered on by every physician in our country. Without implicitly receiving every view contained in the treatise, we cheerfully adopt the closing words of the preface, and heartily commend the work, and the practice therein advocated, "to the candor of that portion of the profession who have the liberality to admit that improvements in the practice of our art can be made; and the energy and honesty to test such proposed improvements before condemning them."

